# Identification of Slc6a19os and SOX11 as Two Novel Essential Genes in Neuropathic Pain Using Integrated Bioinformatic Analysis and Experimental Verification

**DOI:** 10.3389/fnins.2021.627945

**Published:** 2021-01-28

**Authors:** Peng Chen, Chen Wang, Dongsheng Lin, Bing Li, Shuai Ye, Jinglian Qu, Wenjing Wang

**Affiliations:** ^1^Basic Medical School, Guizhou University of Traditional Chinese Medicine, Guiyang, China; ^2^First Clinical Medical School, Guangzhou University of Chinese Medicine, Guangzhou, China; ^3^School of Life Sciences and Technology, Tongji University, Shanghai, China; ^4^School of Life Sciences, East China Normal University, Shanghai, China

**Keywords:** neuropathic pain, SOX11, lncRNA Slc6a19os, ceRNA, miRNA

## Abstract

The aim of this study was to identify critical genes associated with neuropathic pain. We also used the competing endogenous RNA (ceRNA) hypothesis to identify related long non-coding RNAs (lncRNAs) and messenger RNAs (miRNAs) with potential regulatory roles. We downloaded GSE107180 from the Gene Expression Omnibus database, screened differentially expressed genes (DEGs) using R software, performed comprehensive bioinformatic analyses, and validated the expression of lncRNA Slc6a19os, miR-125a-5p, miR-125b-5p, miR-351-5p, and Sox11 by qRT-PCR and Western blots. We identified 620 DEGs in spared nerve injury (SNI) mice compared with sham (control) mice, including 309 mRNAs and 311 non-coding RNAs. The up-regulated mRNAs were enriched primarily in several inflammation-related GO biological processes and KEGG signaling pathways. A ceRNA network was constructed that included 82 mRNAs, 4 miRNAs, and 2 lnRNAs. An ingenuity pathway analysis (IPA)-based interaction network for mRNAs differentially expressed in the ceRNA identified several biological processes, including “cellular development, connective tissue development and function, tissue development.” Compared with sham mice, lncRNA Slc6a19os and Sox11 expression were significantly up-regulated in dorsal root ganglion (DRG) samples from SNI mice detected using qRT-PCR and Western blots (*P* < 0.05). MiR-125a-5p, miR-125b-5p, and miR-351-5p expression were down-regulated in DRG samples from SNI mice detected using qRT-PCR (*P* < 0.05). We concluded that Sox11 and lncRNA Slc6a19os were novel essential genes in the pathogenesis and progression of neuropathic pain and speculated that these two genes were regulated by miR-125a-5p, miR-125b-5p, and miR-351-5p.

## Introduction

Neuropathic pain is defined as pain resulting from a lesion or disease that affects the somatosensory nervous system and is considered a major form of chronic pain (Jensen et al., 2011). Based on global epidemiological studies, the prevalence of neuropathic pain in the general population has been estimated to range from 6.9 to 10% (van Hecke et al., 2014). Neuropathic pain occurs following numerous diseases, including diabetes, spinal cord injury, stroke, postherpetic neuropathy, multiple sclerosis, cancer, infection, trauma, and the toxic effects of chemotherapeutic agents (Alles and Smith, 2018). The main clinical manifestations of this disease include allodynia (pain elicited by stimulus that typically does not produce pain), hyperalgesia (increased pain response produced by a noxious stimulus), and spontaneous pain that commonly accompanies mood disorders such as depression and anxiety, which produces adverse effects on patients' health and life quality (Jensen and Finnerup, 2014; Yalcin et al., 2014).

Currently, the widely recognized mechanisms underlying neuropathic pain are ectopic discharge, peripheral sensitization, central sensitization, abnormal activation of glial cells, and impairment of inhibitory pathways. Despite intense ongoing research, the underlying molecular mechanisms on neuropathic pain remains unclear. The primary pharmacologic agents used to treat neuropathic pain are anti-epilepsy anti-anxiety, and opioid drugs, which have significant adverse side effects, and their analgesic effects often are not satisfactory (Finnerup et al., 2015; Kamerman et al., 2015; Sommer, 2015). Therefore, it is imperative in pain research to investigate the pathogenesis of neuropathic pain in more detail and develop novel, more effective interventions.

Genomic microarrays and bioinformatic analyses have gradually become extremely efficient and convenient methods to identify essential genes involved in neuropathic pain, including long non-coding RNAs (lncRNAs), microRNAs (miRNAs), and messenger RNAs (mRNAs). Wang et al. reported that miR-6838-5p, FZD1, and IL22RA1 might be potential biomarkers that predict spinal cord injury-induced neuropathic pain as well as potential targets for new treatments (Wang et al., 2018). Yu et al. explored the neuroimmune mechanism of neuorpathic pain using bioinformatic analysis and identified CD68, CTSS, LAPTM5, FGR3A, CD53, and HCK as potential diagnostic biomarkers and therapeutic targets (Yu et al., 2020). Also, Tang et al. predicted that mmu-mir-16-5p and MEF2A were essential regulators, and Npy and Atf3 might serve as prognostic and therapeutic genes in neuropathic pain (Tang et al., 2020). However, these studies failed to explore lncRNA ceRNA networks in neuropathic pain, and most of the previously identified targets require further experimental verification.

In this study, we downloaded GSE107180 from the Gene Expression Omnibus (GEO) database, screened differentially expressed genes (DEGs) using R software, performed comprehensive bioinformatic analyses and validated the expression levels of the identified DEGs using animal experimentation. The primary purpose of this study was to identify the essential genes for neuropathic pain and the related lncRNAs and miRNAs that exhibited potential regulatory roles based on the ceRNA hypothesis.

## Methods

### Gene Expression Profile Data Acquisition

The neuropathic pain RNA sequencing dataset, GSE107180, was downloaded from the GEO database (https://www.ncbi.nlm.nih.gov/geo/). GSE107180 included 11 dorsal root ganglion (DRG) samples from sham (control) C57BL/6 mice and 9 DRG samples from spared nerve injury (SNI) C57BL/6 mice.

### Spared Nerve Injury (SNI) Model

The SNI model was established as previously described (Bourquin et al., 2006). Eleven male C57BL/6 mice were randomly divided into the sham (control) group (*n* = 5) and SNI group (*n* = 6). Mice in each group were deeply anesthetized with pentobarbital. The left sciatic nerve of each mouse in the SNI group were exposed, and the tibial and common peroneal nerves were ligated and transected, leaving the sural nerves intact. The left sciatic nerve in each control mouse was exposed but did not undergo any additional procedures. Samples of ipsilateral L3-L5 lumbar DRG were collected after completing behavioral tests at 28 days postoperatively.

### DEGs Identification

The DESeq2 (version 1.26.0) algorithm was used to analyze the raw count matrix in GSE107180 with default parameters to identify DEGs with a threshold of |log2(FC)| ≥1 and *P* < 0.05. DEGs were divided into mRNAs and lncRNAs based on annotations from the mm10 genome (downloaded from UCSC). A volcano plot and heat maps were constructed using R ggplot2 (version 3.3.2) and Complexheatmap (version 2.2.0) packages to display differentially expressed mRNAs and lncRNAs.

### Functional Enrichment Analyses of DEGs

GO and KEGG pathway enrichment analyses of the differentially expressed mRNAs were performed using R package clusterProfiler (version 3.14.3, Yu et al., 2012). The results were ranked by their *P*-value, and the top 10 most significantly enriched biological processes and KEGG pathways were selected.

### Construction of a ceRNA Network

The target miRNAs for all differentially expressed mRNAs in GSE107180 were predicted using the miRWalk database (http://mirwalk.umm.uni-heidelberg.de/) with the default parameters. The target miRNAs for all differentially expressed lncRNAs and mRNAs were predicted using the miRDB database (http://mirdb.org/miRDB/). The intersection of the predicted miRNAs of differently expressed lncRNAs and mRNAs was selected in the two databases described above. The differentially expressed mRNAs and lncRNAs and the intersection of the predicted miRNAs were used to construct a ceRNA network using Cytoscape software (version 3.7.2).

### Ingenuity Pathway Analysis (IPA)

An online integrated software IPA (www.ingenuity.com) was used to analyze the interaction networks of differentially expressed mRNAs involved in the ceRNA network. Briefly, the datasets, including the gene identifiers and their corresponding expression fold changes, were uploaded to the IPA software, and interaction networks with scores that were greater than two were selected and displayed.

### Mechanical Withdrawal Threshold (MWT) Behavioral Test

The mechanical withdrawal threshold (MWT) was measured using a pain threshold detector preoperatively at day 1 and postoperatively at days 3, 7, 14, and 28. Each mouse was placed on the metal mesh floor of a transparent plexiglass box. After a 30-min adaptation period, the von Frey filaments were used to stimulate the lateral surface of the left hind paw of the mouse three times at a 3-min interval with increasing intensity. Lifting or licking indicated a positive response. Then data were recorded and the MWT was determined to be the average response.

### Real-time Quantitative Fluorescence PCR (qRT-PCR)

The ipsilateral L3 to L5 DRGs were collected immediately after completion of the behavioral tests at 28 days post-surgery and the expression of lncRNA Slc6a19os, miR-125a-5p, miR-125b-5p, miR-351-5p, and SOX11 were analyzed using qRT-PCR. Total RNA isolations were carried out using TRIzol reagent (Ambion, Austin, Texas, USA) according to the manufacturer's instructions. The total RNA was converted to cDNA using HiScript Reverse Transcriptase (Vazyme, Nanjing, China) and the following reaction conditions: 25°C for 5 min, 50°C for 15 min, 85°C for 5 min, and 4°C for 10 min. qRT-PCR was conducted using SYBR Green Master Mix (Vazyme, Nanjing, China) and the reaction conditions were set as follows: 95°C for 10 min, 95°C for 15 s, and 60°C for 60 s, and 95°C for 15 s, 60°C for 60 s, and 95°C for 15 s. U6 and β-actin were utilized as internal controls. The primers used in our experiments are listed in Table 1.

**Table 1:** Primer sequences.

**Genes**	**Forward/loop primer**	**Reverse/F primer**
U6	5′-CTCGCTTCGGCAGCACA-3′	5′-AACGCTTCACGAATTTGCGT-3′
β-actin	5′-CACGATGGAGGGGCCGGACTCATC-3′	5′-TAAAGACCTCTATGCCAACACAGT-3′
Slc6a19os	5′-CCTTTCACACCTGCTGCTTAT-3′	5′-AACTCTCCCCATCCTTACCC-3′
Sox11	5′-AGTTCGCCTCCAGCCAGT-3′	5′-TGCGTGTCCACCTCCTCA-3′
miR-125a-5p	5′-GTCGTATCCAGTGCAGGGTCCGAGGT ATTCGCACTGGATACGACTCACAGGT-3′	5′-TGCGCTCCCTGAGACCCTTTAACC-3′
miR-125b-5p	5′-GTCGTATCCAGTGCAGGGTCCGAGGT ATTCGCACTGGATACGACTCACAAGT-3′	5′-TGCGCTCCCTGAGACCCTAACT-3′
miR-351-5p	5′-GTCGTATCCAGTGCAGGGTCCGAGGT ATTCGCACTGGATACGACCAGGCTCA-3′	5′-TGCGCTCCCTGAGGAGCCCTTTGA-3′

### Western Blot Analysis

Protein was extracted from the DRGs using RIPA lysis buffer containing phenylmethylsulfonyl fluoride (PMSF) and phosphatase inhibitor (1:100). The protein concentrations were quantified using a bicinchoninic acid (BCA) protein assay kit (Beyotime, Beijing, China) according to the manufacturer's instructions. The extracted protein supernatant was mixed with the protein loading buffer and boiled at 100°C for 10 min to denature the protein. Then the protein samples were separated on 12% SDS-PAGE gels and transferred to polyvinylidene difluoride (PVDF) membranes (0.45 μm, Millipore, Billerica, Massachusetts, USA). The membranes were incubated overnight at 4°C in rabbit anti-SOX11 (1:500, ABclonal, Wuhan, China) and mouse anti-actin (1:100, ABclonal, Wuhan, China). Then the membranes were incubated with the corresponding secondary antibodies (1:50,000, Boster Biological Technology, Wuhan, China). After washing with tris-buffered saline with tween (TBST), the PVDF membranes were treated with enhanced chemiluminescent (ECL) reagent (Thermo Fisher Scientific, Waltham, MA, USA) and exposed to X-ray film.

### Statistical Analyses

All quantitative data were expressed as means ± standard deviation (SD). Comparisons between the two groups were performed using two-sample *t*-tests. *P* < 0.05 was considered to indicate a statistically significant difference.

## Results

### Identification of DEGs in SNI-Induced Neuropathic Pain

**Figure 1:**
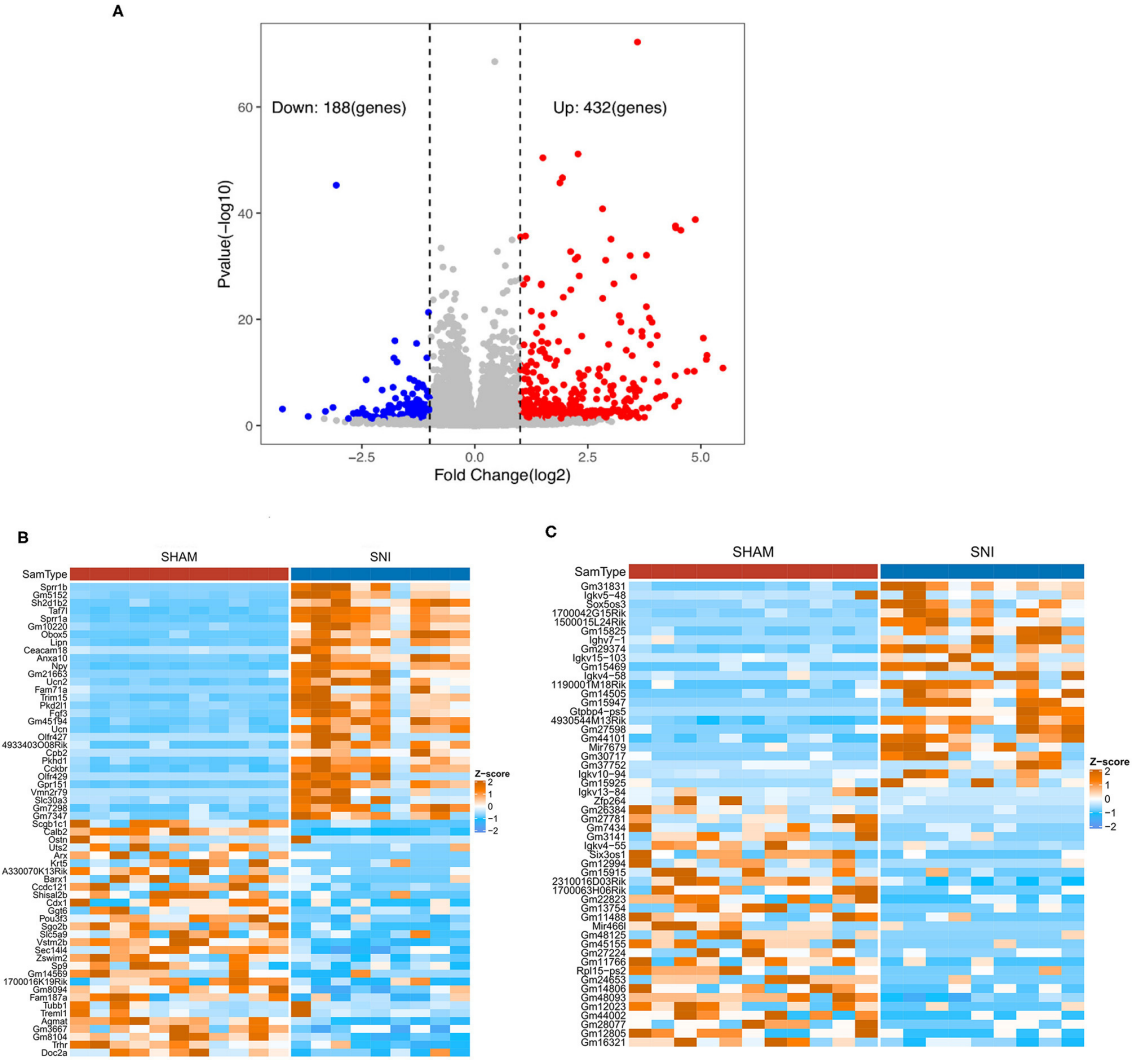
Volcano plot and heat maps of the DEGs obtained from DRG samples from sham and SNI mice. **(A)** The volcano plot of DEGs of DRG samples obtained from sham and SNI mice. **(B)** The heat map showing the top 60 differentially expressed mRNAs from the DRG samples obtained from sham and SNI mice. **(C)** The heat map showing the top 60 differentially expressed lncRNAs obtained from DRG samples from sham and SNI mice.

Based on the criteria for GSE107180 described previously, 620 DEGs were screened between the sham and SNI mice. Compared with the sham mouse DRG samples, we identified 432 up-regulated DEGs, including 254 coding genes and 178 non-coding RNAs, and 188 down-regulated DEGs, including 55 coding genes and 133 non-coding RNAs in SNI mouse samples. The corresponding volcano plot and cluster heat map for the mRNAs and lncRNAs are seen in Figures 1A–C.

### Functional Enrichment Analyses for the DEGs

**Figure 2:**
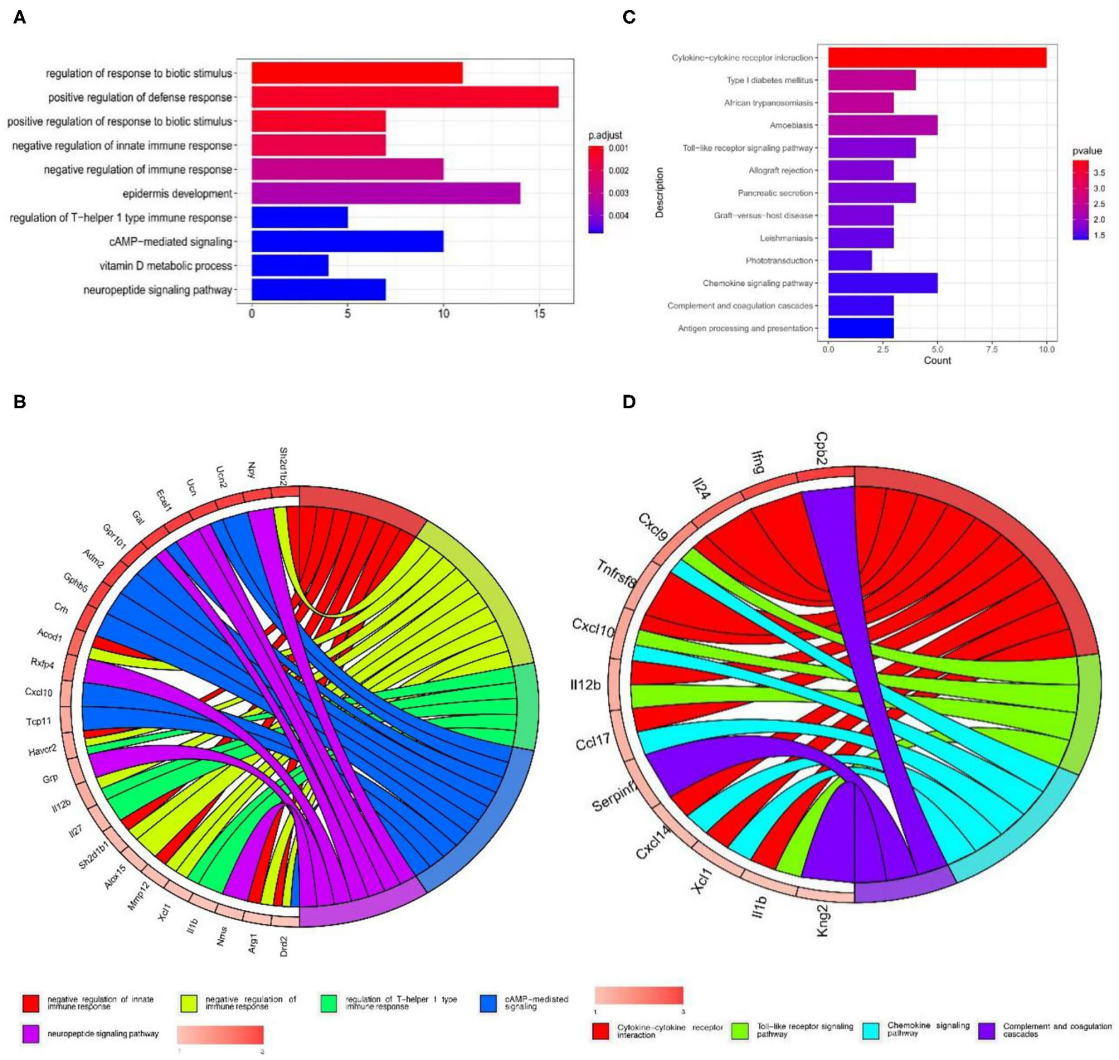
GO and KEGG pathway enrichment analysis of the up-regulated mRNAs in GSE107180. **(A,B)** GO functional enrichment analyses of the up-regulated mRNAs. **(C,D)** KEGG pathway enrichment analysis of the up-regulated mRNAs.

GO annotations and KEGG pathways were performed to determine the functions of the 309 differentially expressed mRNAs. The top 10 enriched biological processes for the up-regulated mRNAs primarily involved regulation of response to biotic stimulus, positive regulation of defense response, positive regulation of response to biotic stimulus, negative regulation of innate immune response, negative regulation of immune response, epidermis development, regulation of T–helper 1 type immune response, cAMP–mediated signaling, vitamin D metabolic process, neuropeptide signaling pathway (Figures 2A,B). The down-regulated mRNAs were not significantly enriched in biological processes of GO annotations.

The top 10 enriched KEGG pathways from the up-regulated mRNAs were selected and showed in Figure 2C. Some of these pathways were associated with neuropathic pain, including cytokine-cytokine receptor interaction, toll-like receptor signaling pathway, chemokine signaling pathway and complement and coagulation cascades (Figures 2C,D).

### Construction of the ceRNA Network

**Figure 3:**
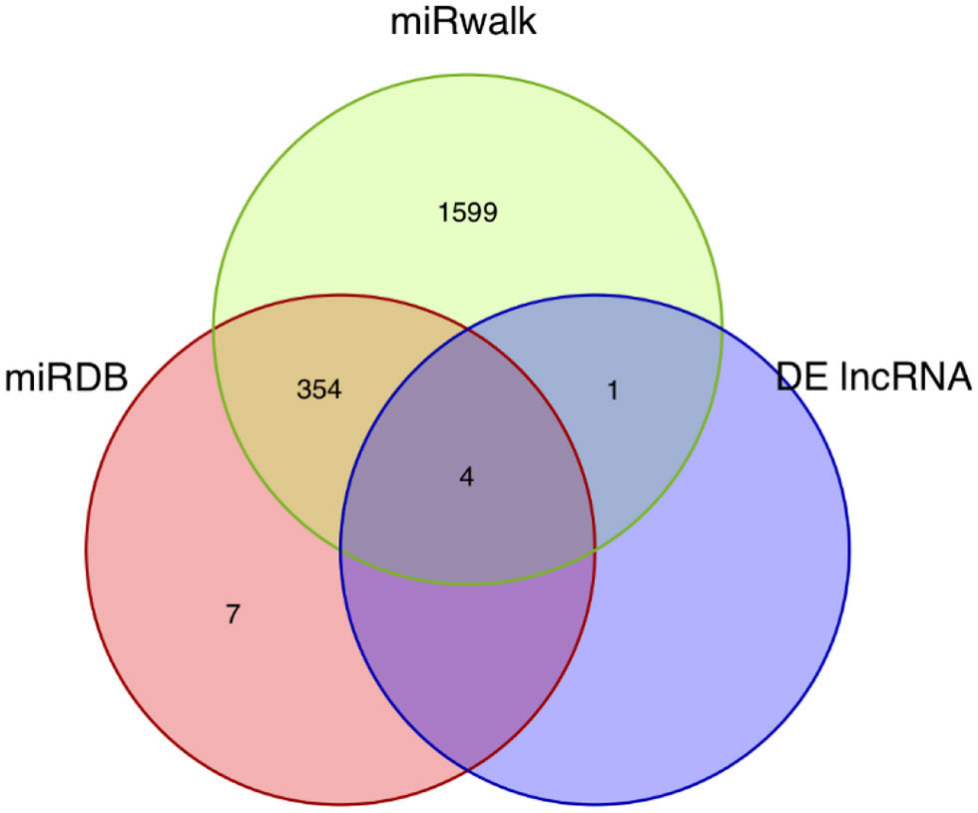
Venn diagram of the miRNA intersections. The Venn diagram shows the intersections of the miRNAs predicted by mRNAs and lncRNAs using the miRDB and miRWalk databases.

62,998 miRNA-mRNA pairs were predicted by the up-regulated mRNAs in the miRWalk database including 1,953 miRNAs and 222 mRNAs. 7,728 miRNA-mRNA pairs were obtained based on down-regulated mRNAs in the miRWalk database, including 1,684 miRNAs and 46 mRNAs. 1,319 miRNA-mRNA pairs were predicted based on the up-regulated mRNAs in the miRDB database and consisted of 360 miRNAs and 166 mRNAs. Two hundred thirty-nine miRNA-mRNA pairs were obtained from the down-regulated mRNAs in the miRDB database and contained 164 miRNAs and 34 mRNAs. Five lncRNA-miRNA pairs were predicted based on the differentially expressed lncRNAs in the miRDB database and included five miRNAs and two lncRNAs. Finally, four common miRNAs were obtained based on the intersection of the predicted miRNAs, including mmu-miR-351-5p, mmu-miR-1197-3p, mmu-miR-125b-5p, and mmu-miR-125a-5p (Figure 3).

**Figure 4:**
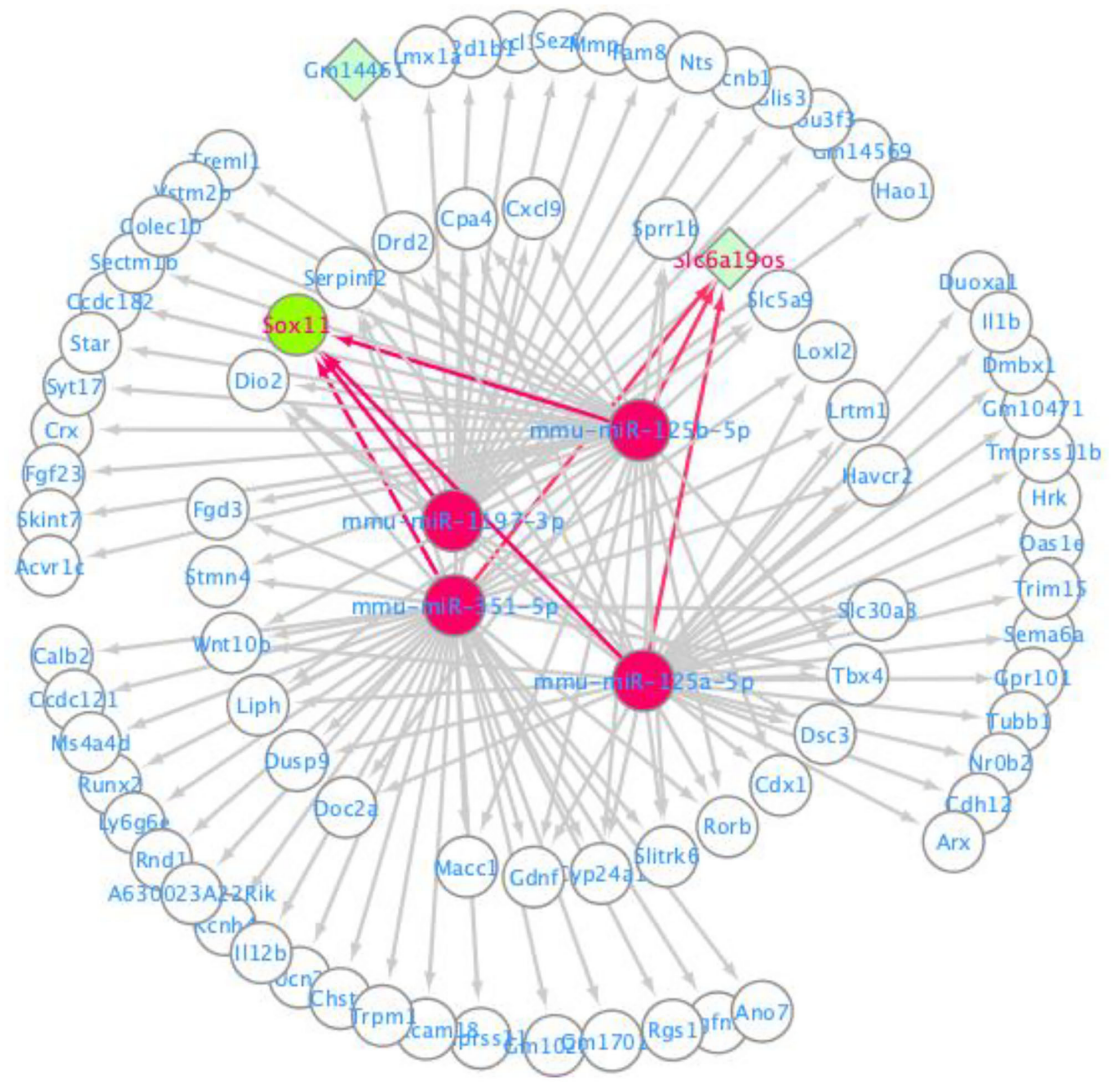
CeRNA regulatory network for lncRNA-miRNA-mRNA.

The ceRNA network was visualized using the Cytoscape software based on the miRNA-mRNA and lncRNA-miRNA regulatory relationships. In this ceRNA network, 88 nodes, including 82 mRNAs, 4 miRNAs and 2 lnRNAs were identified, which indicated that they might be essential regulators of neuropathic pain. These mRNAs and lncRNAs were regulated by mmu-miR-351-5p, mmu-miR-1197-3p, mmu-miR-125b-5p, and mmu-miR-125a-5p (Figure 4).

### Ingenuity Pathway Analysis (IPA)

**Figure 5:**
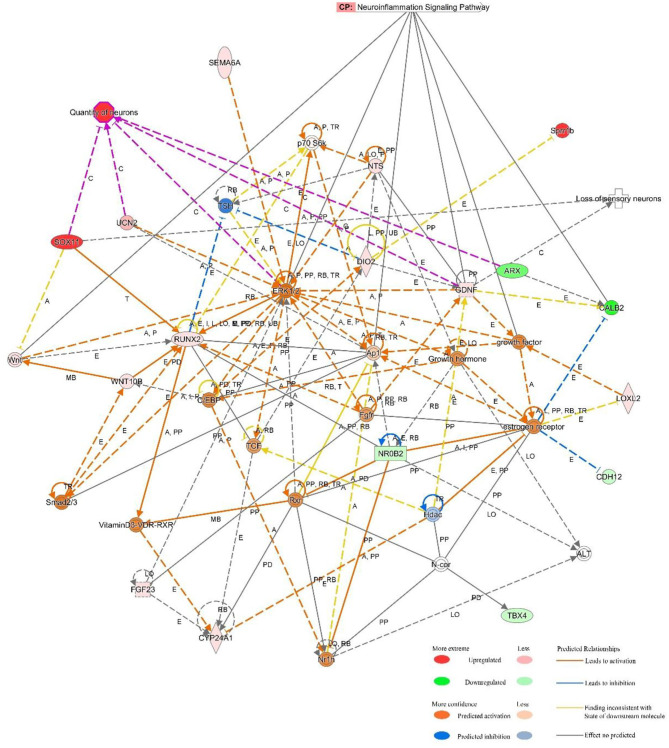
IPA-based interaction networks for the differentially expressed mRNAs involved in “cellular development, connective tissue development and function, tissue development”.

IPA software was used to predict the interacting molecular network to analyze the interaction relationships of the differentially expressed mRNAs involved in the ceRNA network. The results demonstrated that these mRNAs were related to numerous molecular and cellular functions, and particularly enriched in the top three categories, which were cellular development, cardiovascular system development and function, and cellular compromise (Supplementary Figure 1). The top gene-interaction network was “cellular development, connective tissue development and function, tissue development” with a score of 36 and with 17 focus molecules (Figure 5).

### Mechanical Withdrawal Threshold (MWT)

**Figure 6:**
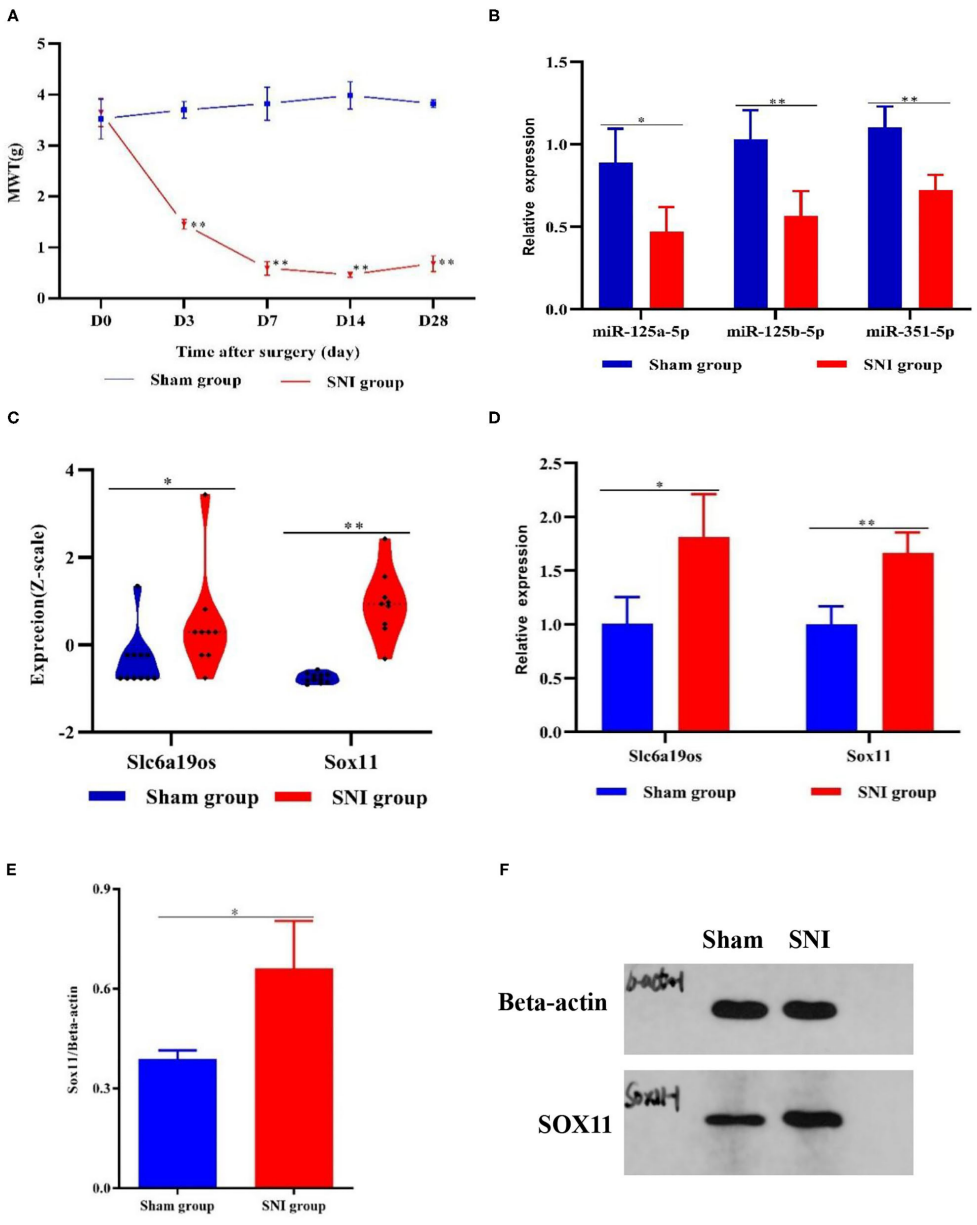
MWT and DEGs in the DRG samples from the sham and SNI mice. **(A)** MWT in the sham and SNI mice. **(B,D)** The expression of Slc6a19os, miR-125a-5p, miR-125b-5p, miR-351-5p, and Sox11 in DRG samples from the sham and SNI mice that were detected using qRT-PCR. **(C)** The expression of Slc6a19os and Sox11 in the DRG samples from the sham and SNI mice in GSE107180. **(E,F)** The expression of Slc6a19os and Sox11 in the DRG samples from the sham and SNI mice that were assessed using Western blot. *P < 0.05 and **P < 0.01 vs. sham.

The MWT was utilized to assess mechanical allodynia in both experimental groups. No significant differences were observed between the two groups initially. However, beginning at the 3rd days after surgery, MWT was dramatically decreased and maintained at lower levels in the SNI mice compared with the sham mice (*P* < 0.01). These results suggested that SNI produced profound mechanical allodynia (Figure 6A).

### Expression of Slc6a19os, SOX11, miR-125a-5p, miR-125b-5p, and miR-351-5p

Research has shown that SOX11 is associated with nervous system development and function. We found that SOX11 and lncRNA Slc6a19os were regulated by three identical miRNAs, miR-125a-5p, miR-125b-5p, and miR-351-5p, in the ceRNA regulatory network and also up-regulated in GSE107180. To ascertain the applicability of the ceRNA network, we validated the lncRNA Slc6a19os-miRNAs-SOX11 pathway. We utilized qRT-PCR and Western blot to verify the expression of lncRNA Slc6a19os, SOX11, miR-125a-5p, miR-125b-5p, and miR-351-5p in SNI mice. Compared with the sham mice, the expression of lncRNA Slc6a19os and SOX11was significantly up-regulated in DRG samples taken from the SNI mice (*P* < 0.05). However, miR-125a-5p, miR-125b-5p and miR-351-5p expression was down-regulated (*P* < 0.05) (Figures 6B–E).

## Discussion

Neuropathic pain is a form of intractable pain that causes tremendous suffering for patients. Currently, the molecular mechanisms of neuropathic pain are unknown, and few effective drugs are available. Thus, it is critical to study the pathogenesis of neuropathic pain to identify potential targets for new therapeutic interventions. Genomic microarrays and bioinformatic analysis have become an efficient and convenient method to identify essential genes involved in neuropathic pain.

In our study, 620 DEGs were identified in SNI mice compared with sham mice, including 309 mRNAs and 311 non-coding RNAs. The up-regulated mRNAs were mainly enriched in several inflammation-related biological processes and signaling pathways, which revealed the key roles of neuroinflammtion in neuropathic pain.

Competing endogenous RNAs (ceRNAs) are transcripts that regulate other RNA transcripts at the post-transcriptional level by competing for the same miRNA-binding sites (Smillie et al., 2018). Several studies have demonstrated that the ceRNA regulatory network for lncRNA-miRNA-mRNA played an important role in the occurrence and development of neuropathic pain (Li H. et al., 2020; Li P. et al., 2020; Song et al., 2020; Wu et al., 2020). In our study, a ceRNA network was constructed based on miRNA-mRNA and lncRNA-miRNA regulatory relationships. Based on the ceRNA network, we speculated that lncRNA Slc6a19os might act as a ceRNA by sponging miR-125a-5p, miR-125b-5p, and miR-351-5p to regulate SOX11 expression indirectly.

Sry-related high-mobility group box (SOX) transcription factors are critical regulators of cell fate during development in multiple systems (Sarkar and Hochedlinger, 2013; Yang, 2017). SOX11 is one of the members of group C of SOX transcription factors, which is extremely important for neuronal development, as well as differentiation in the mammalian peripheral and central nervous systems (Dy et al., 2008; Kavyanifar et al., 2018; Liu P. P. et al., 2019; Turan et al., 2019). Numerous studies have confirmed that SOX11 is significantly upregulated following nerve or spinal cord injury and involved in neural repair and regeneration (Tanabe et al., 2003; Guo et al., 2014; Li et al., 2018; Feng et al., 2019). In our study, we confirmed increased SOX11 expression in SNI-induced neuropathic pain using integrated bioinformatic analysis and experimental verification. Previous studies suggested that the elevation of SOX11 following nerve injury could enhance long-term activation of brain-derived neurotrophic factor (BDNF) transcription and responsiveness of glial cell line-derived neurotrophic factor (GDNF) receptors (Salerno et al., 2012, Guo et al., 2014, Jankowski et al., 2018). BDNF and GDNF are essential regulators of pain perception transmission (Alles and Smith, 2018; Fernandes et al., 2018; Nencini et al., 2018). Therefore, we speculated that SOX11 was involved in the development of peripheral nerve injury-induced neuropathic pain that occurs during nerve repair.

LncRNA Slc6a19os is located on chromosome 13 on the opposite strand of a long non-coding region of solute carrier family 6 member 19 (SLC6A19). LncRNA Slc6a19os is expressed to varying degrees in normal mouse brain tissue. SLC6A19 encodes a transporter that functions to transport neutral amino acids in a solidum-dependent manner (Seow et al., 2004; Margheritis et al., 2016). In our study, the expression level of lncRNA Slc6a19os was significantly increased in SNI mice indicating this gene might be related to neuropathic pain. We speculated that lncRNA Slc6a19os acted as a ceRNA to up-regulate Sox11 expression.

MicroRNAs (miRNAs) are evolutionally conserved, single-stranded small non-coding RNAs that mediate post-transcriptional gene regulation by binding to the complementary 3′ untranslated region of specific mRNAs (Pu et al., 2019). In this study, we found that SOX11 and lncRNA Slc6a19os were both regulated by miR-125a-5p, miR-125b-5p, and miR-351-5p in the ceRNA network. We also confirmed the low expression of predicted miR-125a-5p, miR-125b-5p, and miR-351-5p in the ipsilateral DRG samples of neuropathic pain in SNI mice. Numerous studies have demonstrated that miR-125a-5p was functionally related to immune cell activation and neuroinflammation (Liu Q. et al., 2019; Wang and Guo, 2020). MiR-125b-5p has been reported to attenuate Aβ-induced neurotoxicity in Alzheimer's disease by decreasing apoptosis and oxidative stress in Alzheimer's disease (Li H. et al., 2020; Li P. et al., 2020). Currently, research into the role of miR-351-5p in nervous system disease has been limited. Despite the decrease of miR-125a-5p, miR-125b-5p, and miR-351-5p that are possibly related to the expression levels of SOX 11 and LncRNA Slc6a19os, additional study is needed to determine the exact regulatory relationships of lncRNA-miRNA-mRNA.

Also, IPA was applied to explore the interrelationships of differentially expressed genes in the ceRNA network. We speculated that these genes regulated the expression of extracellular signal-regulated kinase 1/2 (ERK1/2), growth factor, and activating protein 1 (AP1) to mediate neuroinflammation, loss of sensory neurons, and quantity of neurons.

## Conclusions

In summary, we performed an integrated bioinformatics analysis on the GEO database to identify DEGs involved in the pathogenesis of neuropathic pain. We identified SOX11 and lncRNA Slc6a19os as two novel essential genes in the pathogenesis and progression of neuropathic pain and speculated that these genes were regulated by miR-125a-5p, miR-125b-5p, and miR-351-5p.

## Data Availability Statement

The datasets presented in this study can be found in online repositories. The names of the repository/repositories and accession number(s) can be found in the article.

## Ethics Statement

The animal study was reviewed and approved by Animal Use and Ethics Committee, Affiliated Hospital of Guangzhou University of Chinese Medicine.

## Author Contributions

PC, CW, and DL wrote the manuscript. BL, CW, and SY performed the experiments. PC, JQ, and WW conceived of the experiments and analyzed the data. All authors contributed to the article and approved the submitted version.

## Conflict of Interest

The authors declare that the research was conducted in the absence of any commercial or financial relationships that could be construed as a potential conflict of interest.
