# Supplementary figures and images for: Identification of Slc6a19os and SOX11 as Two Novel Essential Genes in Neuropathic Pain Using Integrated Bioinformatic Analysis and Experimental Verification
Source: Front Neurosci. 2021 Jan 28;15:627945. doi: 10.3389/fnins.2021.627945 (PMC7876402; doi:10.3389/fnins.2021.627945)

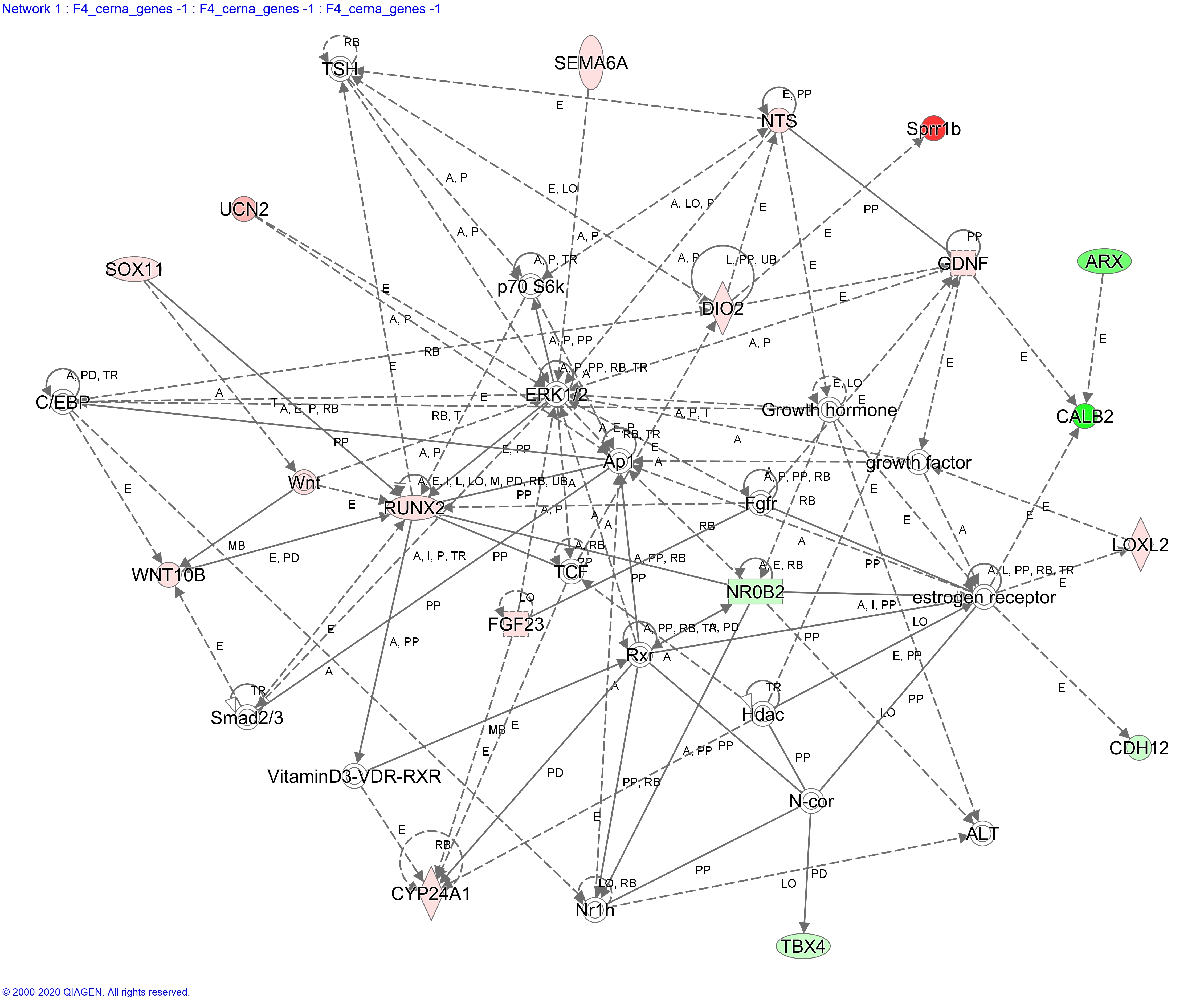

Supplement: Supplementary file 1 [file Image_1.jpg]

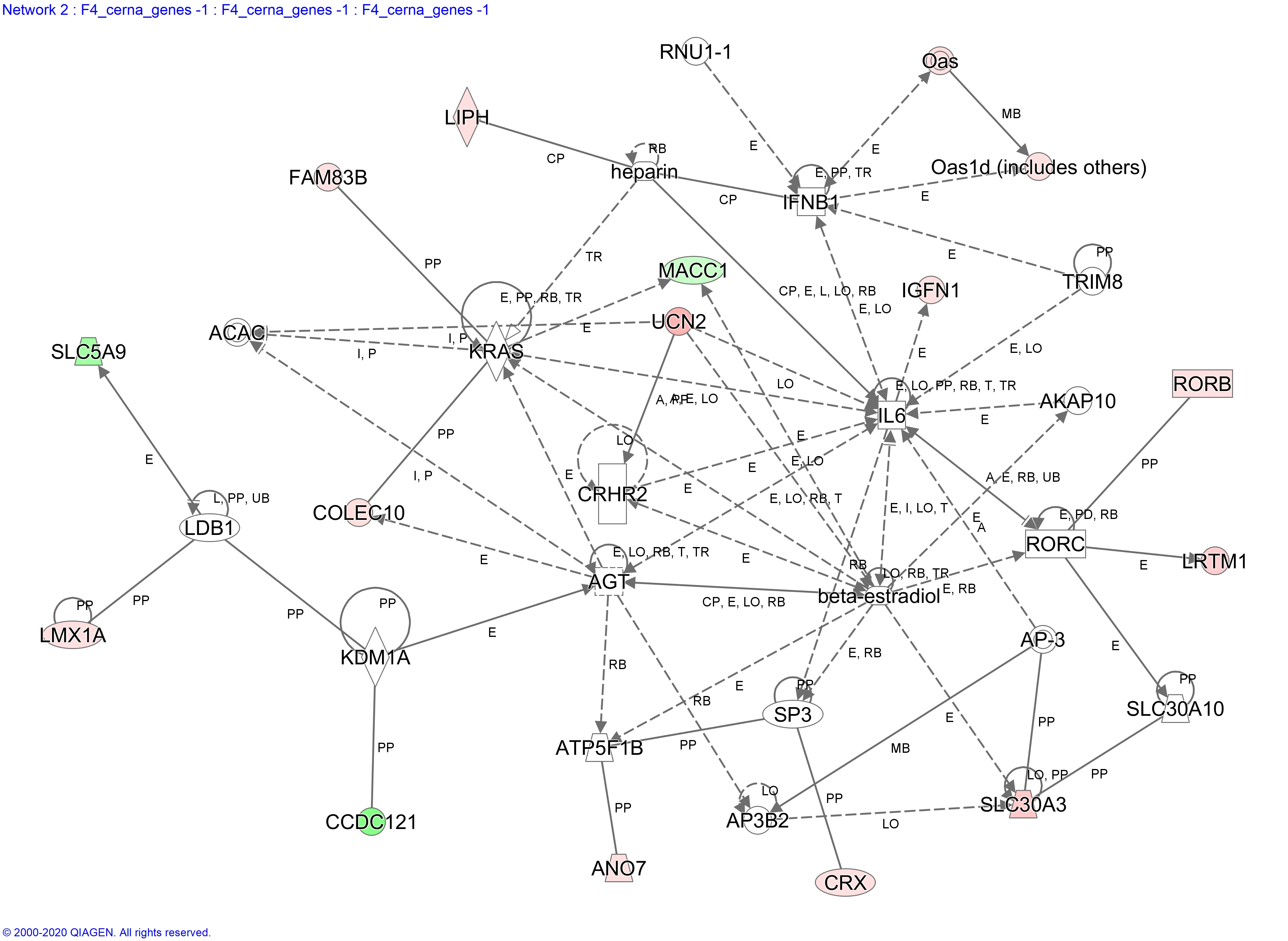

Supplement: Supplementary file 2 [file Image_2.jpg]

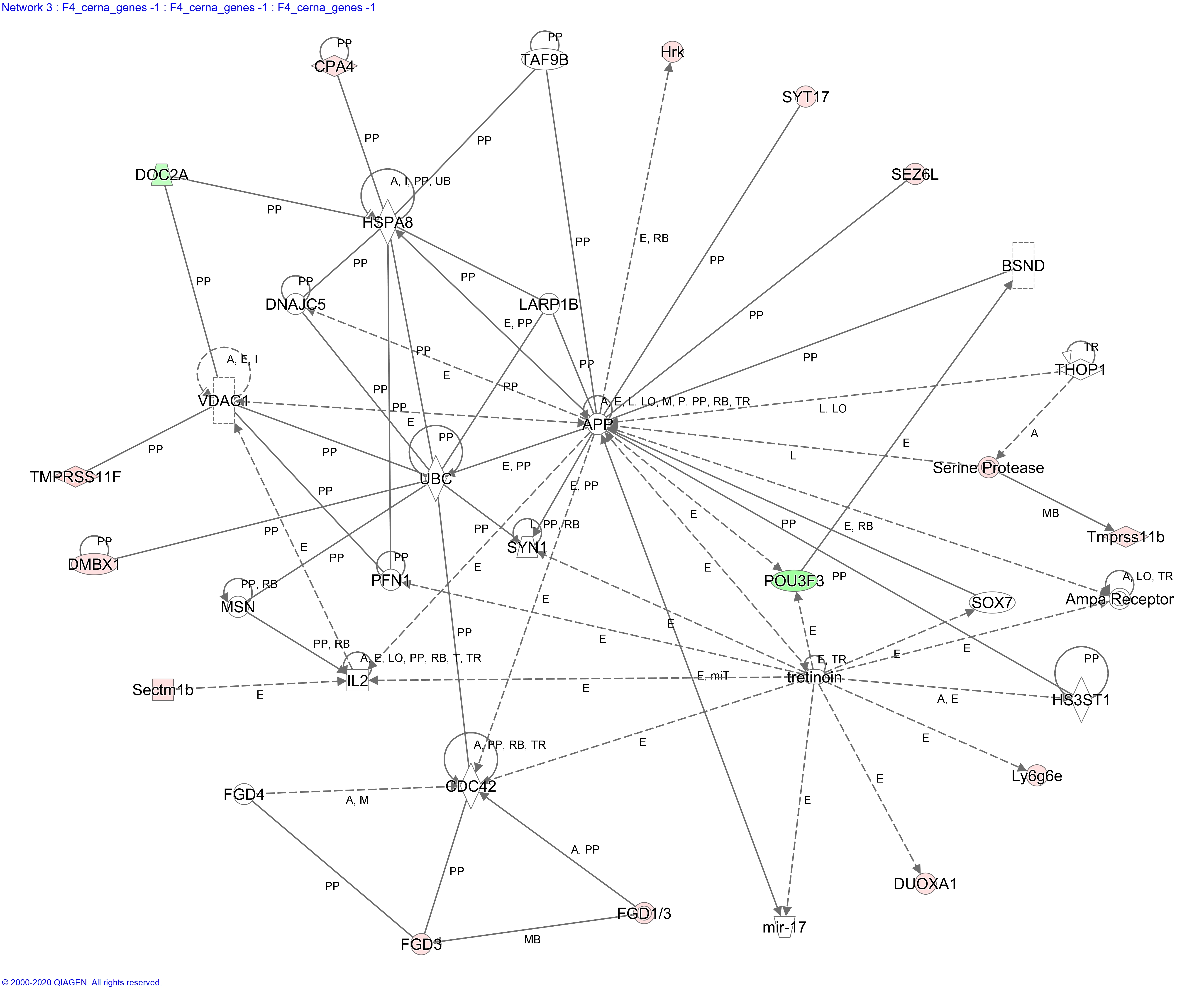

Supplement: Supplementary file 3 [file Image_3.jpg]
